# Chronic Stress Suppresses the Expression of Cutaneous Hypothalamic–Pituitary–Adrenocortical Axis Elements and Melanogenesis

**DOI:** 10.1371/journal.pone.0098283

**Published:** 2014-05-22

**Authors:** Silin Pang, Huali Wu, Qian Wang, Minxuan Cai, Weimin Shi, Jing Shang

**Affiliations:** 1 New Drug Screening Center, China Pharmaceutical University, Nanjing, China; 2 State Key Laboratory of Natural Medicines, China Pharmaceutical University, Nanjing, China; 3 Shanghai First People Hospital, Shanghai, China; University of Tennessee, United States of America

## Abstract

Chronic stress can affect skin function, and some skin diseases might be triggered or aggravated by stress. Stress can activate the central hypothalamic–pituitary–adrenocortical (HPA) axis, which causes glucocorticoid levels to increase. The skin has HPA axis elements that react to environmental stressors to regulate skin functions, such as melanogenesis. This study explores the mechanism whereby chronic stress affects skin pigmentation, focusing on the HPA axis, and investigates the role of glucocorticoids in this pathway. We exposed C57BL/6 male mice to two types of chronic stress, chronic restraint stress (CRS) and chronic unpredictable mild stress (CUMS). Mice subjected to either stress condition showed reduced melanogenesis. Interestingly, CRS and CUMS triggered reductions in the mRNA expression levels of key factors involved in the HPA axis in the skin. In mice administered corticosterone, decreased melanin synthesis and reduced expression of HPA axis elements were observed. The reduced expression of HPA axis elements and melanogenesis in the skin of stressed mice were reversed by RU486 (a glucocorticoid receptor antagonist) treatment. Glucocorticoids had no significant inhibitory effect on melanogenesis in vitro. These results suggest that, high levels of serum corticosterone induced by chronic stress can reduce the expression of elements of the skin HPA axis by glucocorticoid-dependent negative feedback. These activities can eventually result in decreased skin pigmentation. Our findings raise the possibility that chronic stress could be a risk factor for depigmentation by disrupting the cutaneous HPA axis and should prompt dermatologists to exercise more caution when using glucocorticoids for treatment.

## Introduction

Substantial evidence suggests that chronic stress can affect the function of multiple physiological systems, including skin function [1]–[4]. Stress has been associated with the onset and aggravation of many skin disorders, such as psoriasis, alopecia, atopic dermatitis, and vitiligo [5]–[10]. The “brain–skin connection” may underlie skin diseases triggered or aggravated by stress [3], [4], [7], [11].

During exposure to stressful events, the central hypothalamic–pituitary–adrenocortical (HPA) axis is activated. In brief, during stress, corticotropin-releasing hormone (CRH) is synthesized and released, which increases pro-opiomelanocortin (POMC) expression. POMC is converted into adrenocorticotropic hormone (ACTH) and other melanocortin peptides, such as α-MSH. ACTH then binds to the melanocortin type 2 receptor (MC2R) of the adrenal cortex and stimulates glucocorticoid synthesis and secretion into systemic circulation to exert various physiological effects. In the adrenal cortex, P450scc (Gene symbol: CYP11A1) acts as a key enzyme in glucocorticoid synthesis. Glucocorticoid binding to glucocorticoid receptors (GRs) in hypophysiotropic neurons and the anterior pituitary gland can inhibit the release of CRH and ACTH to allow for negative feedback regulation of the HPA axis [12]–[15].

The skin is the largest organ in the human body and can be regulated by the immune and neuroendocrine systems [3], [16]–[18]. Skin reacts to environmental stressors, such as ultraviolet radiation (UVR), in a strikingly similar manner to the activities of the central HPA axis [3], [19], [20]. The skin and its major appendages express key molecules, including CRH or urocortin (UCN1), POMC, and P450scc, along the classical HPA axis. CRH or UCN1 can interact with CRH receptor type 1 (CRHR1) to produce POMC-derived peptides, with the latter stimulating the local production of glucocorticoids [21]–[28]. After exposure to UVR, the gene expression levels of CRH or UCN1, POMC, and CYP11A1 in the skin increase [19], [29]. Simultaneously, the skin is also the target organ of CRH and the related urocortin peptide, POMC-derived peptides, and glucocorticoids [3], [4], [22], [23], [26], [27], [30]. Treating melanocytes with CRH has been shown to induce melanogenesis [22], [26], [31]. POMC, ACTH, and α-MSH also increase melanogenesis in melanocytes [31]–[33]. Thus, skin pigmentation has a close relationship with cutaneous HPA axis activation.

Because of the evolutionary conservation of HPA-like networks at the central and cutaneous levels, interactions may exist between these systems, and such interactions could affect the functions of each systerm [4], [34]. For example, UVR of the skin results in excitation of the central nervous system [3], [35], and skin disorder is associated with higher central HPA axis activity [36]. The possibility of communication between the cutaneous and systemic HPA axes poses an interesting question for research [3]. However, to our knowledge, very few studies have investigated the influence of the higher central HPA axis on the skin. Thus, this study aimed to explore the effects of chronic stress on skin function and to test whether the cutaneous HPA axis is involved. Here, C57BL/6 male mice were subjected to two types of chronic stress, and the effects of these stresses on skin were observed. Because high levels of serum corticosterone were observed in stressed mice, normal mice were administered corticosterone and stressed mice were administered a glucocorticoid receptor antagonist to explore the role of glucocorticoids in the effects of stress on the skin.

## Materials and Methods

### Animals

Male C57BL/6 mice (5 weeks old, 20.6±2.1 g) were obtained from the Laboratory Animal Services Center of the Yangzhou University. Animals were maintained on a 12-h light/dark cycle at a regulated temperature (22±2°C), humidity (50±10%) and fed a standard diet and water ad libitum. Animals were acclimatized for 7 days. This study was carried out in strict accordance with the guidelines of the “Principles of Laboratory Animal Care” (NIH Publication No. 80–23, revised in 1996). This study was specifically approved by the Animal Experimentation Ethics Committee of the Chinese Pharmaceutical University (Approval ID: SCXK - (Su) 2011–0003). All efforts were made to minimize suffering.

### Chronic stress application

Mice were randomly divided into the following three groups: (1) control group, (2) chronic restraint stress (CRS) group, and (3) chronic unpredictable mild stress (CUMS) group. The CRS procedure was performed as described before [37]. The CUMS procedure was performed as described before with adjustments [38]. In brief, CUMS consisted of a variety of unpredictable stressors, namely, 14-h food deprivation, 14-h water deprivation, 3-min swimming, 1-min tail pinch, 0.5 h cage shock, 24-h soiled cage, and overnight illumination. One of these stressors was given every day for 21 days.

### Drugs

Corticosterone was purchased from Sigma-Aldrich (MO, USA). Mice were randomly divided into control and corticosterone (CORT) groups. Mice received corticosterone injections (20 mg/kg, subcutaneously), once per day for 21 days.

RU486 (a glucocorticoid receptor antagonist) was purchased from Sigma-Aldrich. Mice were randomly divided into the following three groups: (1) control group, (2) CUMS group with no drug injection and the application of CUMS, and (3) CUMS+RU486 group with the application of CUMS concomitant with 100 mg/kg/day of RU486 injected subcutaneously for 21 days.

### Depilation and tissue sample collection

**Figure 1:**
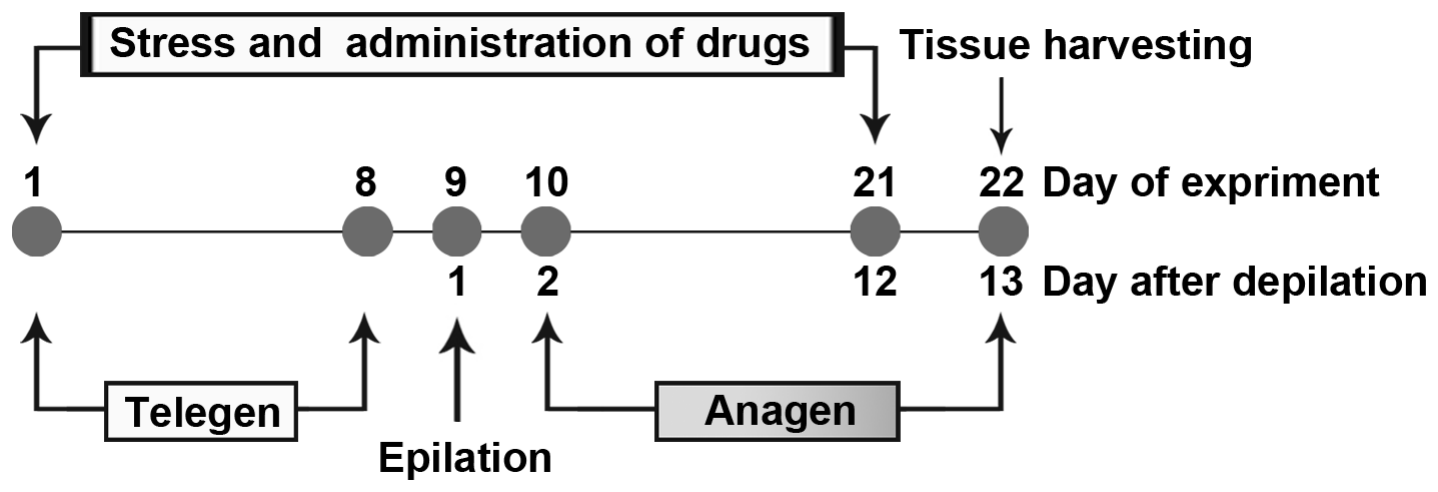
Time table of the experiments. CRS or CUMS was administered beginning on day 1 and continued for 21 days. Drugs were also administered from day 1 for 21 days. All mice received epilation to induce anagen of the hair cycle at day 9. Mice were photographed on days 10 and 22, which were 2 and 13 days post-depilation. Tissue samples were collected on day 22.

On day 9, all mice received epilation with rosin and paraffin (1∶1, w:w) to induce anagen of the hair cycle (Fig. 1) [39]. Mice were maintained under intraperitoneal anesthesia with chloral hydrate (300 mg/kg) during epilation. On day 22, mice were sacrificed after taking blood samples from eyes under anesthesia between 09:00 am and 10:00 am. Blood was incubated at room temperature for 1 h to allow clotting to occur, and serum was then separated by centrifugation and stored at −70°C until use. The back skin of mice was collected and stored in liquid nitrogen until use.

### Cell culture

The B16F10 murine melanoma cells were purchased from the Cell Bank of the Chinese Academy of Sciences. Cell culture was performed as described before [40]. α-MSH and dexamethasone (DEX) were purchased from Sigma-Aldrich (MO, USA). To detect the effects of DEX and α-MSH on melanogenesis, cells were incubated with 1 µM DEX or 50 nM α-MSH. B16F10 cells at passage numbers between 5 and 7 were used for the experiments.

Normal human epidermal melanocytes were derived from young male adult foreskins (ethnic Han/aged 18–22 years) obtained at circumcision following standard protocols [41]. Melanocytes were incubated with MCDB153 medium (Sigma-Aldrich, USA) containing 1 nM choleratoxin (Sigma-Aldrich, USA), 0.1 mM 3-isobutyl-1-methylxanthine (Sigma-Aldrich, USA), 1.6 nM phorbolesters (Sigma-Aldrich, USA), 5 µg/mL insulin (Sigma-Aldrich, USA) and incubated at 37°C in a humidified atmosphere containing 5% CO_2_. To detect the effects of DEX and α-MSH on melanogenesis, cells were incubated with 1 µM DEX or 50 nM α-MSH. Melanocytes at passage numbers between 3 and 6 were used for the experiments. The studies on human material were approved by Nanjing Drum Tower Hospital, Medical Ethics Committee. All participants provided their written informed consent to participate in this study. This consent procedure was approved by the Nanjing Drum Tower Hospital, Medical Ethics Committee.

### Measurement of body weight and corticosterone analysis

The body weight of all mice were recorded on days 3, 6, 9, 12, 15, 18, and 21. Serum corticosterone concentrations were measured using the IBL-AMERICA Corticosterone rat/mouse ELISA kit (IBL, USA) according to the manufacturer's instructions. Serum samples were incubated at room temperature and then directly used for detection. The lowest detectable concentration of corticosterone that could be distinguished from the “zero calibrator” was 4.1 ng/mL.

### Assessment of skin pigmentary response

All mice were photographed with a digital camera (Canon, Japan) once every day after depilation. The grayscale (0–255) of specific area in the photographs (the region from neck to tail) were analyzed by Image J software and presented as ratios (grayscale/255).

### Measurement of melanin contents

Cells were incubated with compounds for 72 hours. After they were washed twice with ice-cold phosphate-buffered saline (PBS), cells were lysed by incubation in cell lysis buffer [20 mM Tris PH 7.5, 150 mMNaCl, 1% TritonX-100, 2.5 mM sodium pyrophosphate, 1 mM EDTA, 1%Na3VO4, 0.5 µg/ml leupeptin, 1 mM phenylmethanesulfonyl fluoride (PMSF)] (Biyuntian, China) at 4°C for 10 min, then the lysates were centrifuged at 14,000 rpm for 15 min. The supernatant containing protein was removed and conserved. Protein concentrations were determined by BCA Protein Assay kit (Biyuntian, China) with bovine serum albumin (BSA) (Sigma-Aldrich, USA) as a standard. The precipitate containing total melanin was dissolved in 100 µL of 1N NaOH/10%DMSO for 2 h at 80°C. Total melanin content was estimated by absorbance at 405 nm and comparison made with total protein concentration, then calculated as a percent of the control.

### Western blotting

The protein suspension of skin tissues was obtained using a Total Protein Extraction Kit (APPLYGEN, China) and the protein concentrations were detected by BCA Protein Assay kit with BSA as a standard. The same amount of total protein was assayed for each Western blot. Proteins were separated by SDS-PAGE and transferred to nitrocellulose membranes. The membranes were incubated with primary antibodies: POMC (1∶200, Santa Cruz Biotechnology Inc, USA) and β-Actin (1∶4000, Sigma-Aldrich, USA) for 1.5 h at room temperature then washed with Tris-buffered saline, including 0.1% Tween-20, exposed to peroxidase-conjugated secondary antibodies (1∶4000, Sigma-Aldrich, USA) for 1 h at room temperature, washed. Proteins were visualized using an enhanced chemiluminescence detection system. Densitometric analysis was performed by using Quantity One (Bio-Rad, USA). Three animals were used for each data point. Western blot assay results reported here are representative of three independent experiments. The related protein bands, cut and displayed in the same figure, are from the same membrane.

### Quantitative real-time PCR

Total RNA was extracted from cells or mouse dorsal skin using TRIZOL reagent (Gibco-BRL, USA) and total RNA concentration was quantified spectrophotometrically. First strand cDNA was synthesized with PrimeScript RT Master Mix (Takara, Japan) according to the manufacturer's instructions. The quantitative real-time PCR was performed on an iQ5 multicolor real-time PCR detection system (Bio-Rad, USA) by using SYBR Premix Ex TaqTM2 (Takara, Japan) according to the manufacturer's instructions. Primer sequences are shown in Table 1. Real-time PCR conditions were: 1 cycle of 2 min at 50°C, 95°C for 10 min, followed by 40 cycles of 95°C cDNA denaturation for 20 s, 60°C primer annealing for 30 s and 72°C extension for 30 s. Melting curve analyses were performed to confirm absence of nonspecific bands. The expression levels of each gene were normalized against β-Actin (Gene symbol: Actb) or GAPDH, then calculated as fold change using the comparative 2^-ΔΔCT^ method and the results were from at least three independent experiments according to the manufacturer's protocols [42].

**Table 1:** Primer sequences.

Genes	Species	Forward (F) and Reverse (R) primer sequences		Product size (bp)
MITF	mouse	F	TGCTCGCCTGATCTGGTGAAT	152
		R	GTGCCGAGGTTGTTGGTAAAGG	
TYR	mouse	F	GATGGAACACCTGAGGGACCACTAT	150
		R	GCTGAAATTGGCAGTTCTATCCATT	
Hsd11b1	mouse	F	CTCCTCCCGATCCTGGTGCTCT	132
		R	TGCCATTTCTCTTCCAATCCCTTT	
Hsd11b2	mouse	F	GTTAACAACGCTGGCCTCAATA	160
		R	CAACGGTCACAATACGTCCCCT	
Nr3c1	mouse	F	GGATGACCAAATGACCCTTCTACAG	112
		R	ATCAGGAGCAAAGCATAGCAGGTT	
CRHR1	mouse	F	CTCACGTACTCCACCGACCG	130
		R	TGCCAAACCAGCACTTTTCA	
CRHR2	mouse	F	CCCTGTGGACACTTTTGGAGC	183
		R	GGGTCGTGTTGTACTTGATGCC	
MC1R	mouse	F	GGCTGTCGTGGGCATCTGGA	204
		R	ATGGACCGCCGCCTTTTGTG	
MC2R	mouse	F	ACCATCATCACCCTAACAAT	136
		R	GACACAGGATAAAAACCAGC	
UCN1	mouse	F	CACTGTCCATCGACCTCACCTTC	117
		R	ACTTGCCCACCGAATCGAATA	
POMC	mouse	F	TTGCTGAGAACGAGTCGGC	86
		R	GACCTGCTCCAAGCCTAATGG	
CYP11A1	mouse	F	AGATGCCTGGAAGAAAGACCGAA	199
		R	GATGGACTCAAAGGAAAAGCGGA	
Actb	Mouse	F	CAGGTCATCACTATTGGCAACGAG	87
		R	GATGCCACAGGATTCCATACCC	
MITF	Human	F	ACGAGAACAGCAACGCGCAAA	145
		R	GCAGAGACCCGTGGATGGAATA	
TYR	Human	F	GTTGCGGTGGGAACAAGAAATC	164
		R	AGAAGAATGATGCTGGGCTGAGTAA	
GAPDH	Human	F	CGCTGAGTACGTCGTGGAGTC	172
		R	GCTGATGATCTTGAGGCTGTTGTC	

### Statistical analysis

Statistical analysis was performed using GraphPadPrismVersion 5.0c (GraphPad Software). Data were analyzed by unpaired, two-tailed Student's t test or by one-way ANOVA with Tukey's post hoc test, as appropriate. P<0.05 was regarded as significant. Results are presented as mean ± SEM.

## Results

### Effects of chronic stress on the mouse dorsal skin melanogenesis

**Figure 2:**
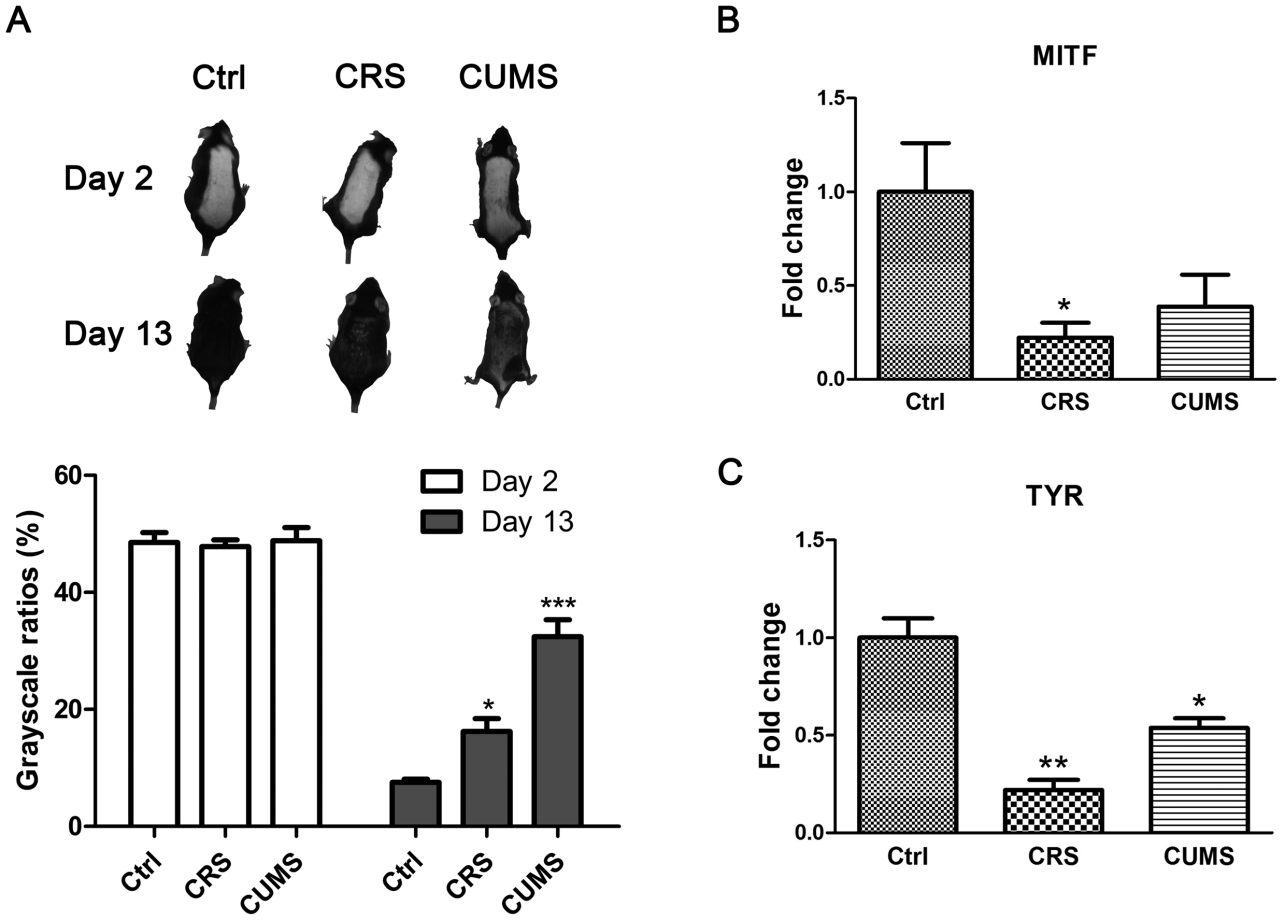
Chronic stress causing reduction of melanogenesis in mice dorsal skin. **A**: Photographs of mice back skin on day 2 and day 13 after epilation showing the reduction of melanin in the skin of CRS and CUMS group mice on day 13. **B**: The mRNA expression levels of microphthalmia-associated transcription factor (MITF) in mouse skin. **C**: The mRNA expression levels of tyrosinase (TYR) in mouse skin. The expression levels of each gene were normalized against β-Actin then calculated as fold change using the comparative 2^-ΔΔ^CT method. Data are showed in mean ± SEM, n = 8, and the data were analyzed by one-way ANOVA with Tukey's post hoc test. * *P<0.05*, ** *P<0.01*, *** *P<0.001*, compared with control.

Mice were photographed after epilation. Upon visual examination, all groups of mice had a similar skin color on the second day after epilation. However, on the thirteenth day, the skin color showed visible differences, and stressed mice showed lighter skin color than control mice (Fig. 2A).

Experiments were performed to detect any changes in microphthalmia-associated transcription factor (MITF) or tyrosinase (TYR) mRNA expression levels in the dorsal skin. MITF is a key transcription factor for melanogenesis that effectively transactivates the TYR genes by binding to an M-box motif in the TYR promoter [43]. Tyrosinase is the rate-limiting enzyme in two critical steps in melanogenesis [44]. The CRS mice showed reduced mRNA expression levels of MITF (P<0.05) and TYR (P<0.01) (Fig. 2B, C). The CUMS mice showed reduced mRNA expression levels of TYR (P<0.05) (Fig. 2C).

### Effects of chronic stress on the cutaneous HPA axis

**Figure 3:**
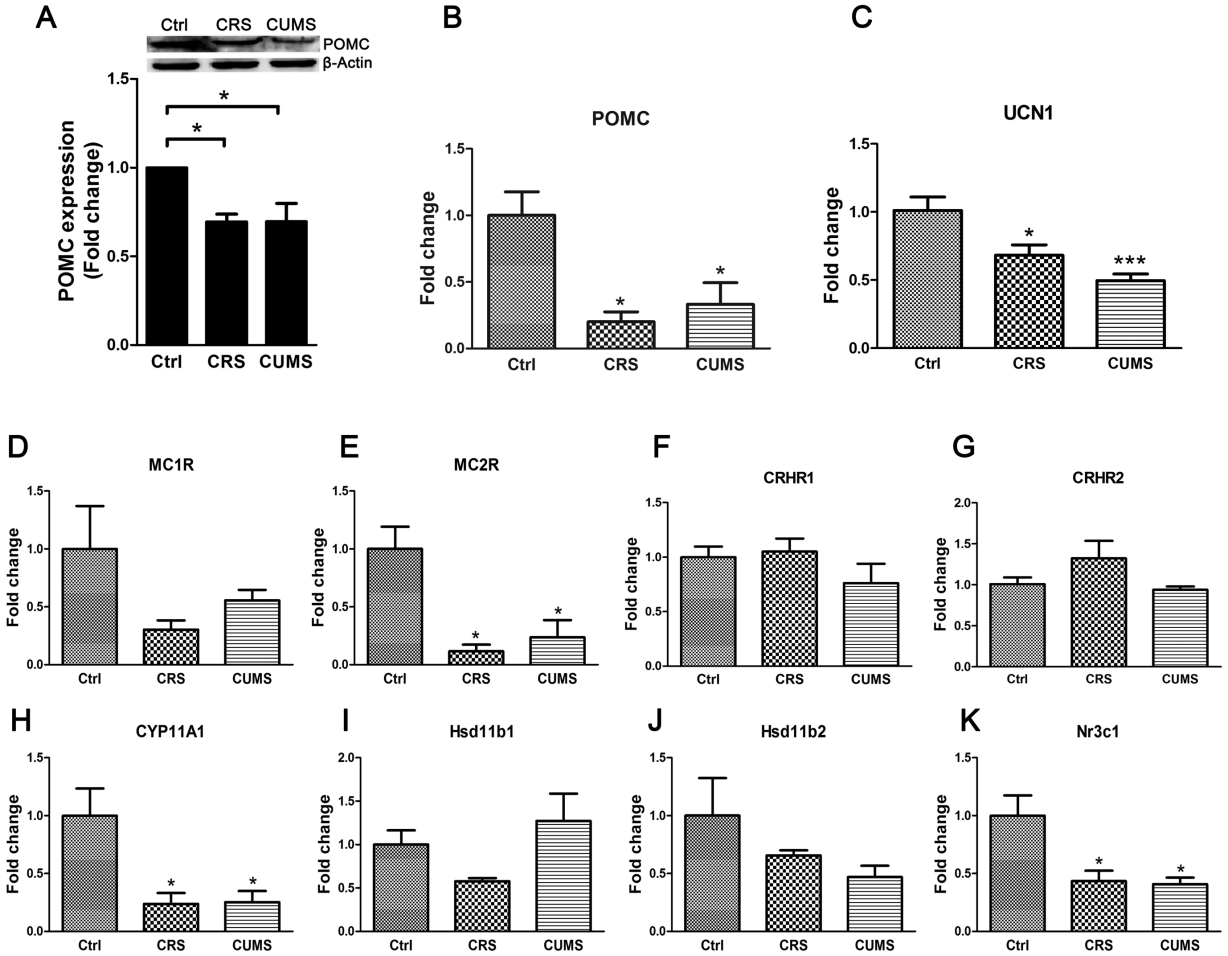
Chronic stress causing disrupted expressions of cutaneous HPA axis elements. **A**: POMC expression was analyzed by immunoblotting. β-Actin expression was indicated as a loading control. Western blot assay are representative of three experiments. Densitometric scanning of band intensities obtained from three separate experiments was used to quantify change of proteins expression. Three animals were used for each data point. Data are showed in mean ± SEM. **B–K**: The mRNA expression levels of POMC, UCN1, MC1R, MC2R, CRHR1, CRHR2, CYP11A1, Hsd11b1, Hsd11b2, and Nr3c1 in mouse skin. The expression levels of each gene were normalized against β-Actin then calculated as fold change using the comparative 2^-ΔΔCT^ method. Data are showed in mean ± SEM, n = 8. Data were analyzed by one-way ANOVA with Tukey's post hoc test. * *P<0.05*, ** *P<0.01*, compared with control.

The expression of POMC was examined in mouse skin. POMC expression levels were reduced by both CRS (P<0.05) and CUMS (P<0.05) (Fig. 3A). Similarly, the mRNA expression levels of POMC in mouse skin were also reduced by CRS (P<0.05) and CUMS (P<0.05) (Fig. 3B). The mRNA expression levels of POMC cleavage product receptors were also measured, and chronic stress reduced MC2R mRNA expression levels (Fig. 3E). Although there were decreased mRNA expression levels of MC1R in the skin of stressed mice, the changes were not significantly different from the levels in control mice (Fig. 3D).

Alterations of POMC expression in the skin of stressed mice may be caused by changes in UCN1 expression, thereby affecting the cutaneous HPA axis, and may lead to changes in the expression of CYP11A1. To test this possibility, experiments were performed to measure mRNA expression of UCN1 and CYP11A1, and both genes were found to be reduced in the skin of stressed mice (Fig. 3C, H). UCN1 exerts biological effects by binding to its receptors [28], [45], [46]. The mRNA expression levels of CRHR1 and CRHR2 did not significantly change in the skin of stressed mice (Fig. 3F, G). 11β-hydroxysteroid dehydrogenase type 1 (11β-HSD1, Gene symbol: HSD11b1), 11β-hydroxysteroid dehydrogenase type 2 (11β-HSD2, Gene symbol: HSD11b2), and glucocorticoid receptor (GR) play key roles in the regulation of glucocorticoid activities [47]–[50]. The expression of the genes that encode 11β-HSD1 (Hsd11b1) and 11β-HSD2 (Hsd11b2) showed no significant changes in the skin of stressed mice, whereas the expression of the gene that encodes GR (Gene symbol: Nc3r1) was reduced (Fig. 3I–K).

### Effects of chronic stress on body weight and serum corticosterone levels

**Figure 4:**
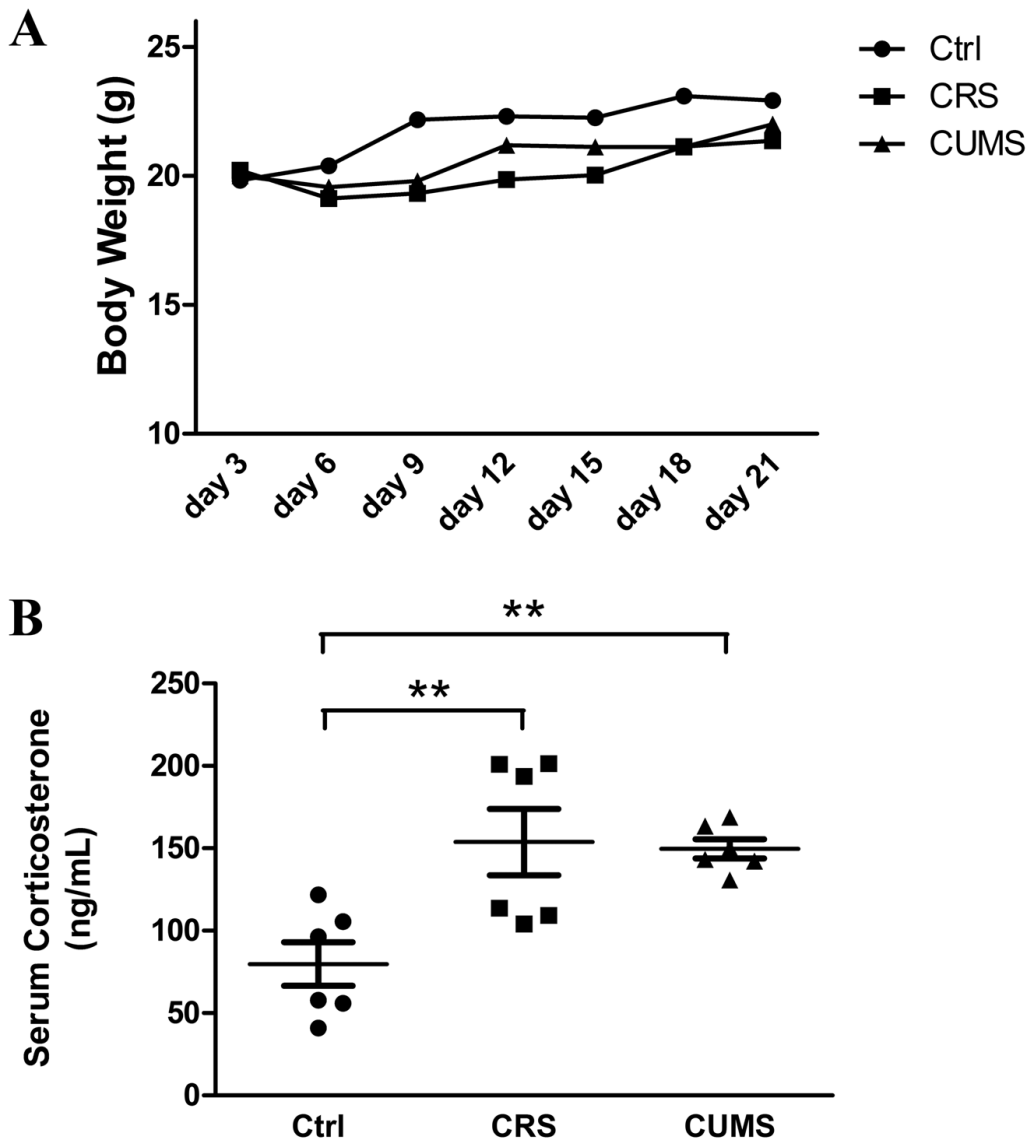
Effects of chronic stress on mice body weight gain and serum corticosterone levels. **A**: On days 3, 6, 9, 12, 15, 18, and 21, CRS and CUMS did not inhibit mice body weight gain significantly compared with control. **B**: The serum corticosterone levels in mice of different group. Serum for corticosterone measurement was collected on day 22, one day after the final stressor. Data are showed in mean ± SEM, n = 6, and the data were analyzed by one-way ANOVA with Tukey's post hoc test. ** *P<0.01*, compared with control.

Our data indicated that CRS and CUMS did not significantly inhibit mouse body weight gain compared to the control group (Fig. 4A).

Sustained activation of the central HPA axis by chronic stress can lead to elevated glucocorticoid levels [51]. Compared to controls (Ctrl, 79.72±12.08 ng/mL), chronic stress caused a significant elevation of serum corticosterone levels (CRS, 153.83±18.36 ng/mL; CUMS, 149.75±5.31 ng/mL) (Fig. 4B).

### Effects of corticosterone on the dorsal skin of mice

**Figure 5:**
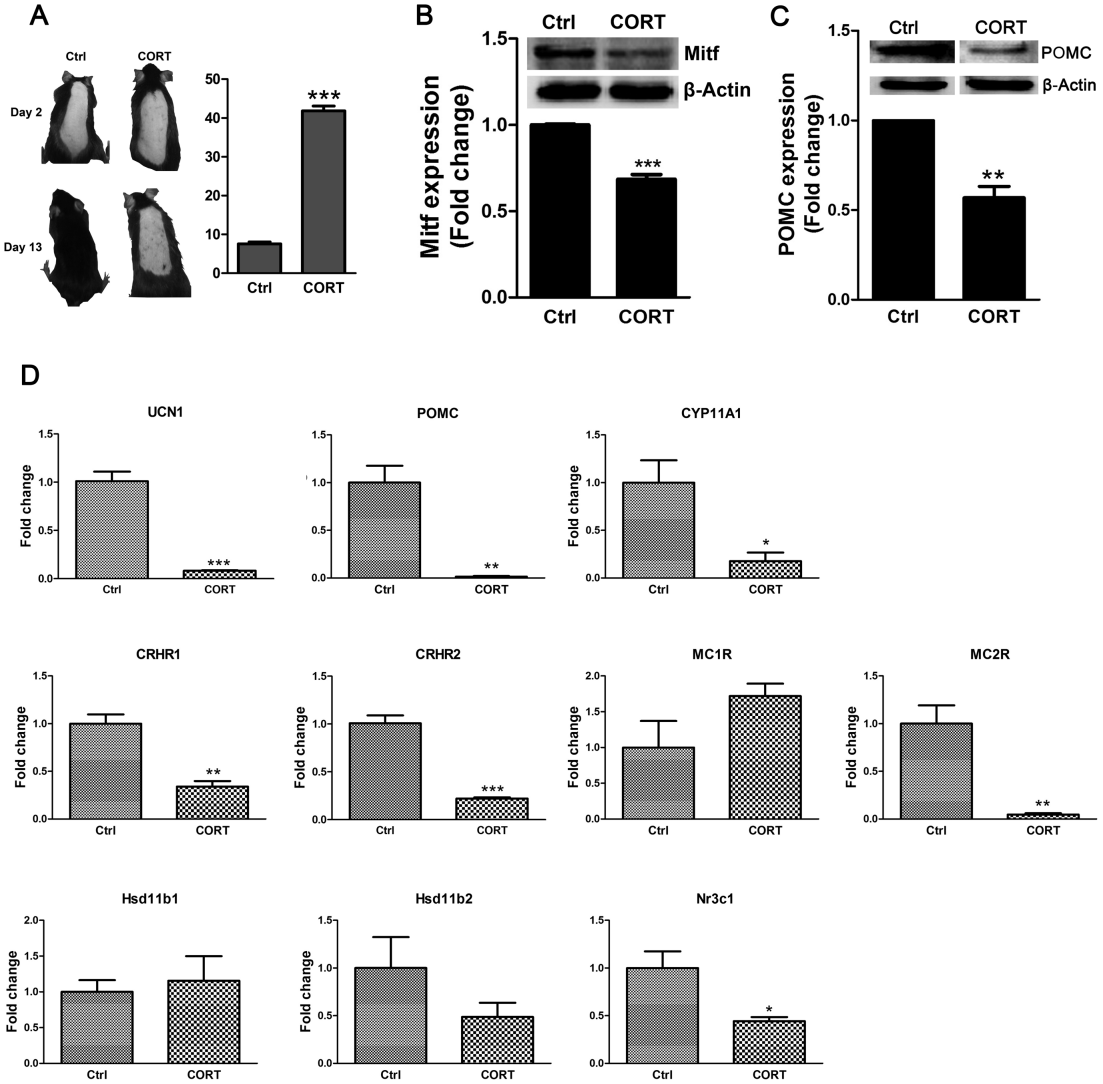
Corticosterone causing reduction of melanogenesis and disrupted expressions of cutaneous HPA axis elements. **A**: Photographs of mice back skin on day 2 and day 13 after epilation showing the reduction of melanin in corticosterone treated mice on day 13. **B**: MITF expression was analyzed by immunoblotting. **C**: POMC expression was analyzed by immunoblotting. β-Actin expression was indicated as a loading control. Western blot assay are representative of three experiments. Densitometric scanning of band intensities obtained from three separate experiments was used to quantify change of proteins expression. Three animals were used for each data point. Data are showed in mean ± SEM. **D**: The mRNA expression levels of UCN1, POMC, CYP11A1, CRHR1, CRHR2, MC1R, MC2R, Hsd11b1, Hsd11b2, and Nr3c1 in mouse skin. The expression levels of each gene were normalized against β-Actin then calculated as fold change using the comparative 2^-ΔΔCT^ method. Data are showed in mean ± SEM, n = 8. Data were analyzed by Student's t test. * *P<0.05*, ** *P<0.01*, *** *P<0.001*, compared with control.

Considering that chronic stress caused a marked elevation of serum corticosterone levels, further studies were conducted to investigate whether corticosterone affects the dorsal skin of mice in vivo. On the thirteenth day after epilation, mice that received corticosterone injections had a significantly lighter observable skin color than controls (Fig. 5A). In the CORT group, mice had significantly lower MITF expression (Fig. 5B). These results suggest that corticosterone reduces skin melanogenesis.

The expression levels of key factors along the HPA axis were evaluated in the dorsal skin of mice that received chronic corticosterone injections. POMC expression was reduced after corticosterone injections (Fig. 5C). Corticosterone also significantly suppressed mRNA expression levels of UCN1, POMC, CYP11A1, MC2R, CRHR1, CRHR2 and Nr3c1 (Fig. 5D).

### Effects of RU486 on the dorsal skin of stressed mice

**Figure 6:**
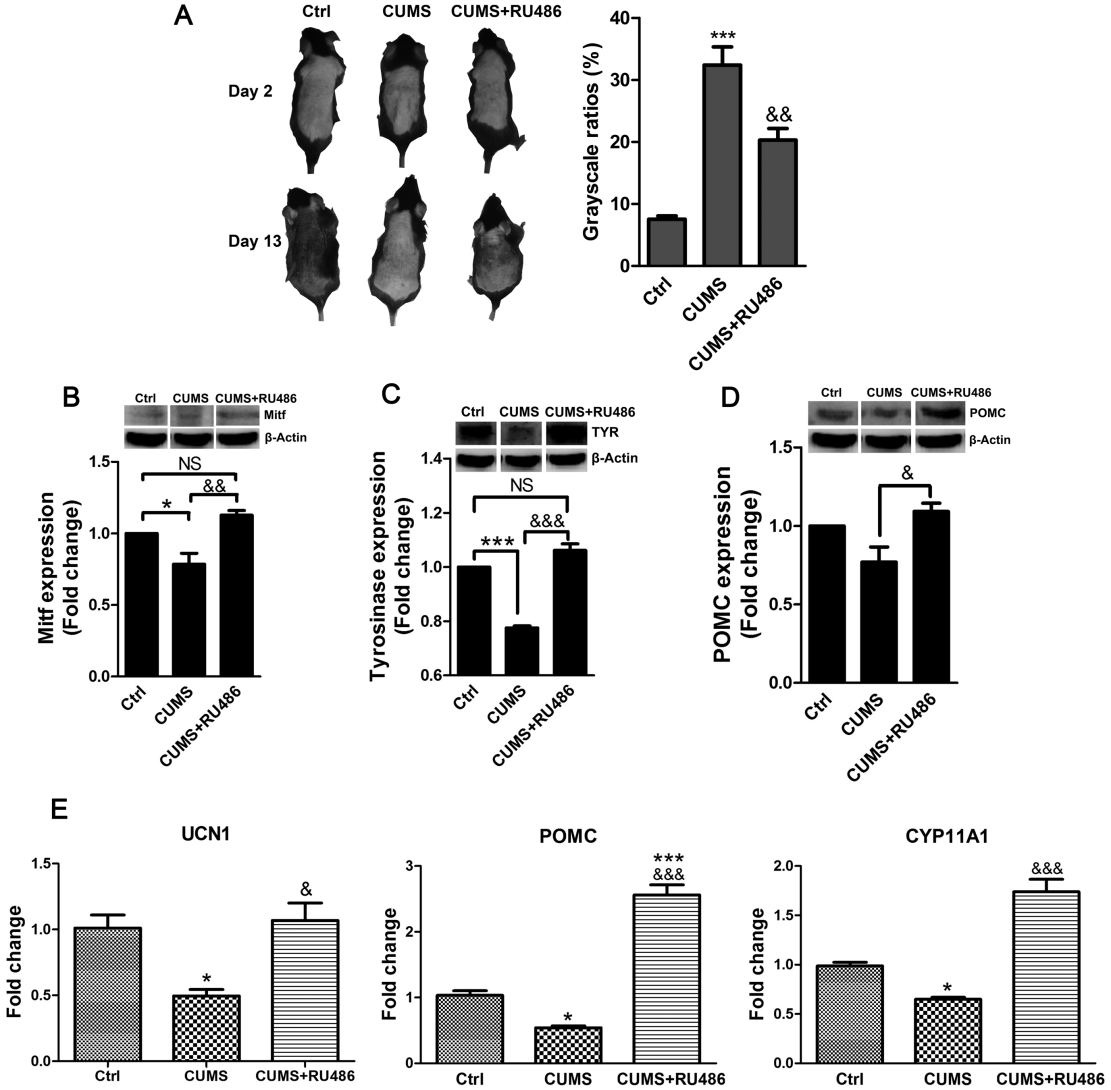
Effects of RU486 on the dorsal skin of stressed mice. **A**: Photographs of mice back skin on day 2 and day 13 after epilation showing that the mice received RU486 injection had a significantly darker observable skin color than CUMS mice. **B**: MITF expression was analyzed by immunoblotting. **C**: TYR expression was analyzed by immunoblotting. **D**: POMC expression was analyzed by immunoblotting. β-Actin expression was indicated as a loading control. Western blot assay are representative of three experiments. Densitometric scanning of band intensities obtained from three separate experiments was used to quantify change of proteins expression. Three animals were used for each data point. Data are showed in mean ± SEM. **E**: The mRNA expression levels of UCN1, POMC, and CYP11A1 in mouse skin. The expression levels of each gene were normalized against β-Actin then calculated as fold change using the comparative 2^-ΔΔCT^ method. Data are showed in mean ± SEM, n = 8. The data were analyzed by one-way ANOVA with Tukey's post hoc test. * *P<0.05*, *** *P<0.001*, compared with control; ^&^ *P<0.05*, ^&&^ *P<0.01*, ^&&&^ *P<0.001*, compared with CUMS.

Because chronic stress caused a marked elevation of serum corticosterone levels, further studies were conducted to test whether RU486 (a glucocorticoid receptor antagonist) could reverse the effects of CUMS on the dorsal skin of mice in vivo. On the thirteenth day after epilation, mice that received RU486 injections had a significantly darker observable skin color than CUMS mice (Fig. 6A). The CUMS mice showed reduced expression of MITF and TYR, but RU486 reversed this effect (Fig. 6B, C). Additionally, administration of RU486 significantly ameliorated the CUMS-induced reduction of POMC expression (Fig. 6D). Furthermore, RU486 also normalized the CUMS-induced reduction of mRNA expression levels of UCN1, POMC, and CYP11A1 (Fig. 6E). These results suggested that stress-induced alterations of these mediators might be mediated by glucocorticoids.

### Effects of HPA axis-related hormones on melanin synthesis in vitro

**Figure 7:**
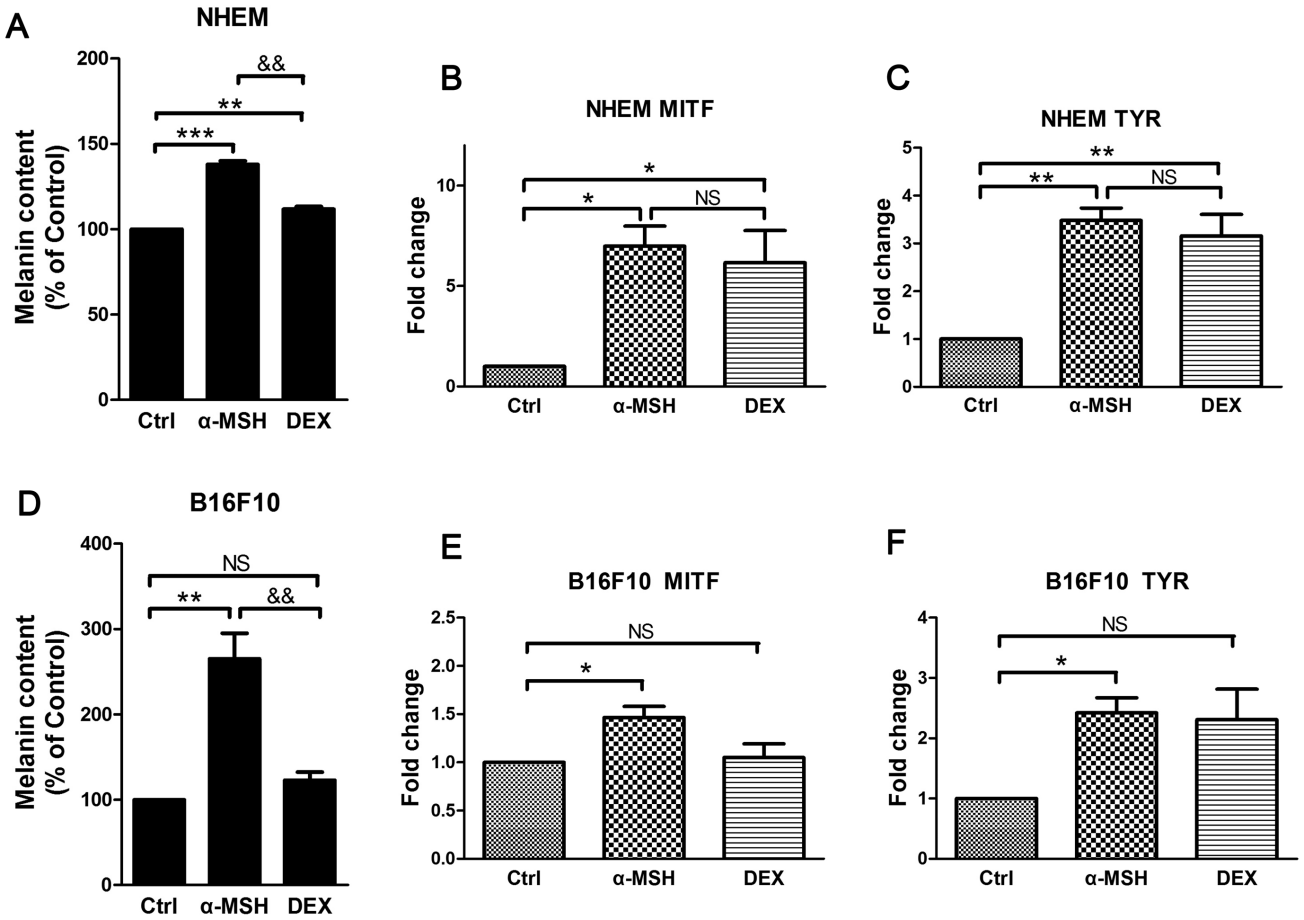
Effects of HPA axis-related hormones on melanin synthesis in vitro. **A**: Measurement of melanin contents in normal human epidermal melanocytes (NHEMs) after treatment with 50 nM α-MSH or 1 µM DEX for 72 h. **D**: Measurement of melanin contents in B16F10 cells after treatment with 50 nM α-MSH or 1 µM DEX for 72 h. **B–C**: The mRNA expression levels of MITF and TYR in NHEMs after treatment with 50 nM α-MSH or 1 µM DEX for 24 h. **E–F**: The mRNA expression levels of MITF and TYR in B16F10 cells after treatment with 50 nM α-MSH or 1 µM DEX for 24 h. The expression levels of each gene were normalized against β-Actin or GAPDH then calculated as fold change using the comparative 2^-ΔΔCT^ method. Data are combined from three separate experiments and showed in mean ± SEM, and the data were analyzed by one-way ANOVA with Tukey's post hoc test. * *P<0.05*, ** *P<0.01*, *** *P<0.001*, compared with control; ^&&^ *P<0.01*, compared with α-MSH.

To determine whether corticosterone could directly inhibit melanogenesis, NHEMs or B16F10 cells were incubated with α-MSH or DEX, a synthetic corticosteroid. In NHEMs, both α-MSH and DEX significantly increased melanin content, and α-MSH showed a stronger effect than DEX (Fig. 7A). Next, we used RT-PCR to examine the effects of these hormones on the expression of genes associated with melanogenesis. Both α-MSH and DEX increased mRNA expression levels of MITF and TYR, and again α-MSH showed a stronger effect than DEX (Fig. 7B, C). In B16F10 cells, α-MSH significantly increased the melanin content, whereas DEX showed no significant effect (Fig. 7D). We found that α-MSH increased the mRNA expression levels of MITF and TYR, whereas DEX showed no significant effect (Fig. 7E, F). These results suggested that glucocorticoids showed no inhibitory effects on melanogenesis in B16F10 cells and that glucocorticoids promoted melanogenesis in NHEMs.

## Discussion

Our data demonstrate that chronic stress can suppress cutaneous melanogenesis and the expression levels of cutaneous HPA axis elements. Moreover, chronic stress can cause the elevation of serum corticosterone levels, suggesting that increased corticosterone levels may contribute to the suppression of chronic stress. Consistent with this possibility, mice that received subcutaneous injections of corticosterone showed reduced expression of cutaneous HPA axis elements and decreased pigmentation. Additionally, the glucocorticoid receptor antagonist increased the CUMS-induced reductions of melanogenesis and cutaneous HPA axis element expression levels. These findings suggest that stress can suppress the activation of the cutaneous HPA axis through glucocorticoids and thereby cause reduced melanogenesis.

In a study of human hair follicles in vitro, the glucocorticoid receptor agonist hydrocortisone could reduce follicular CRH expression [22]. The skin of DEX-treated mice shows attenuated production of POMC mRNA [52]. These data, along with our observations that subcutaneous injections of corticosterone can reduce the expression of HPA axis hormones in the skin of mice, suggest that the skin has HPA axis-like regulatory feedback systems that are mediated by glucocorticoids. Stress is the main factor that drives increased central HPA axis activation [1]. Both CRS and CUMS cause sustained responsiveness of the central HPA axis [53], [54] and caused high levels of serum glucocorticoids in our study. These results imply that stress may attenuate the activation of the cutaneous HPA axis. Some researchers have reported that 2 h restraint stress treatment could suppress cutaneous POMC mRNA expression levels [55]. Together with the inhibitory effect of chronic stress on the cutaneous HPA axis, it is possible that this phenomenon is caused by glucocorticoid-dependent negative feedback.

In addition to the decreased expression of hormones along the cutaneous HPA axis in situ, chronic stress also leads to changes in the expression of cognate receptors for these hormones. Mice that experienced chronic stress or long-term corticosterone injections exhibited reduced MC2R mRNA expression levels in skin. ACTH binds to MC2R to promote glucocorticoid synthesis [56]. Reduced expression of MC2R and POMC may cause a reduction in glucocorticoid synthesis, which is consistent with the reduced expression of CYP11A1 observed in our experiments. CRHR1 and CRHR2 are important receptors of the HPA axis. Hypothalamic CRHR1 gene transcription in mice has been shown to be inhibited by glucocorticoid administration [56]. Corticosterone-treated mice had decreased cutaneous expression levels of CRHR1 and CRHR2, whereas stressed mice showed no significant differences. Substance P can increase the expression of CRHR1 in mast cells in human skin [57]. There is increased Substance P protein expression in cutaneous peripheral nerve fibers in chronically stressed mice [58]. The involvement of other factors that increase in response to stress in skin might underlie the differential expression of CRHR1 and CRHR2 in chronically stressed and corticosterone-treated mice.

Based on our findings, glucocorticoids play an important role in the regulation of chronic stress. In humans, two key enzymes that regulate local cortisol availability are 11β-HSD1 and 11β-HSD2, which induce intracellular conversion of cortisol, and, together with GR, play a key role in regulating glucocorticoid activities [47]–[50]. Expression of 11β-HSD1, 11β-HSD2, and GR has been detected in human skin [59], [60]. We found that the mRNA expression levels of HSD11b1 and HSD11b2 could be detected in the skin of mice and that the expression level of these genes was not affected by chronic stress or corticosterone injections. Recent studies have shown that chronic stress can reduce GR expression in the brain, and this effect may be mediated by elevated glucocorticoid levels [61], [62]. We found that both chronically stressed and corticosterone-treated mice showed reduced mRNA expression of Nr3c1. This phenomenon may be caused by desensitization of skin exposed to high glucocorticoid concentrations.

human skin expresses mRNAs for three obligatory enzymes of steroid synthesis including cytochromes P450scc, P450c17 and P450c21 [63]. P450scc also shows pleiotropic effects in cutaneous secosteroidal system [3], [64], [65]. Since chronic stress and corticosterone treatment suppressed mRNA expression levels of CYP11A1, we propose that chronic stress and glucocorticoids treatment may affect steroidogenesis and secosteroidogenesis in the skin.

Clinically, glucocorticoids are used to treat many skin diseases [66]–[68]. Although the precise role of the cutaneous HPA axis in skin function remains to be determined, the multiple functions of HPA axis-derived hormones on the skin adds further evidence to the role of the cutaneous HPA axis in maintaining homeostasis in the skin microenvironment [11], [20], [69]. In addition to some direct effects of glucocorticoids on skin [68], [70], [71], local glucocorticoid treatment may affect skin function by restraining the activation of the cutaneous HPA axis. Moreover, skin disorder is accompanied by psychological pressure, which can lead to elevated cortisol levels [2]. Based on our data, we speculate that these patients could have attenuated expression of cutaneous HPA axis elements. Our findings should prompt dermatologists to be more cautious when using glucocorticoids for treatment.

Melanogenesis is an important function of the skin that protects the body against radiation and helps maintain homeostasis of the skin microenvironment [33], [72], [73]. Melanogenesis is closely related to activation of the skin HPA axis. In patients with depigmentation, decreased epidermal POMC processing and α-MSH levels have been previously reported [74]. In this study, we found no significant inhibitory effect of glucocorticoids on melanogenesis in B16F10 cells. In NHEMs, glucocorticoids could promote melanogenesis, however this effect was weaker than α-MSH treatment. However, administering corticosterone to mice resulted in reduced melanin synthesis, which is consistent with a previous report that the application of DEX to the skin of mice decreased tyrosinase protein concentration [52]. The inhibitory effect of corticosterone and chronic stress on melanogenesis in vivo appears to be indirect. The reduced pigmentation may be caused by the repressed expression of skin HPA axis elements, which are caused by glucocorticoids. Taking into account that glucocorticoids promotes melanin synthesis and that glucocorticoids exert their biological effects by binding to their receptor, the reduced expression of glucocorticoid receptor in chronically stressed and corticosterone-treated mice may have also caused the reduced pigmentation.

In summary, our data demonstrate that chronic stress can suppress the expression of skin HPA axis-related genes and proteins. This restriction of the cutaneous HPA axis may be caused by negative feedback control via high glucocorticoid concentrations induced by stress. Additionally, the suppressed expression of elements of the cutaneous HPA axis is accompanied by reduced pigmentation, emphasizing that chronic stress may be a risk factor for the development of skin problems.
